# Association of smoked and smokeless tobacco use with migraine: a hospital-based case–control study in Dhaka, Bangladesh

**DOI:** 10.1186/1617-9625-11-15

**Published:** 2013-07-05

**Authors:** Mohammad Abul Bashar Sarker, Mahbubur Rahman, Md Harun-Or-Rashid, Shaila Hossain, Hideki Kasuya, Junichi Sakamoto, Nobuyuki Hamajima

**Affiliations:** 1 Young Leaders’ Program in Healthcare Administration, Nagoya University Graduate School of Medicine, Nagoya, Japan; 2 Center for Interdisciplinary Research in Women’s Health, Department of Obstetrics and Gynecology, University of Texas Medical Branch, Galveston, USA; 3 Institute of Public Health (IPH), Mohakhali, Dhaka, Bangladesh; 4 Community Medicine Department, National Institute of Preventive and Social Medicine (NIPSOM), Mohakhali, Dhaka, Bangladesh; 5 Department of Surgery II, Nagoya University Graduate School of Medicine, Nagoya, Japan; 6 Tokai Central Hospital, Tokai, Japan

**Keywords:** Migraine, Smoked tobacco, Smoking, Smokeless tobacco, Bangladesh

## Abstract

**Background:**

Several studies in the past have reported inconclusive evidences on association of smoking and migraine. Nevertheless, no study so far reported association of smokeless tobacco with migraine. The objective of this study was to examine the association of smoked and smokeless tobacco use with migraine.

**Methods:**

A hospital-based case–control study was conducted at the neurology outpatient department of a tertiary care hospital in Dhaka, Bangladesh. We enrolled 138 migraine cases diagnosed during March-September 2010 in neurology outpatient department, and 276 gender and age matched healthy controls from among their attendants. Diagnosis of migraine was based on the International Headache Society criteria. Use of smokeless tobacco and smoking (cigarette/bidi/hukka) were determined by an interviewer administered questionnaire.

**Results:**

Among the cases, 52.9% were overall tobacco users; 24.6% were only smokers, 15.9% only smokeless tobacco users and 12.3% used both. The respective figures among controls were 14.5%, 7.2%, 6.9% and 0.4% (*P* <0.001 for all). The conditional logistic regression analysis found that migraine had higher odds of exposure to smoked tobacco use, smokeless tobacco use, and both compared to control after adjusting for confounding variables (alcohol drinking, insufficient sleep, mental stress, and number of family members); adjusted odds ratio (aOR) was 6.6 (95% confidence interval [CI] = 2.2-19.6, *P* = 0.001), 5.8 (95%CI = 1.9-17.4, *P* = 0.001), and 54.2 (95%CI = 4.3-684.4, *P* = 0.002), respectively. The aOR of cigarette/bidi/hukka smoking for different doses was 5.5 (95%CI = 1.2-24.8, *P* = 0.027) for 1–5 times per day, 6.3 (95%CI = 1.8-21.2, *P* = 0.003) for 6–10 times per day, and 6.7 (95%CI = 1.9-23.2, *P* = 0.003) for >10 times per day relative to non users.

**Conclusions:**

Both smoked and smokeless tobaccos were found to be associated with migraine. There is a need to incorporate smokeless tobacco along with smoked tobacco into the anti-tobacco awareness programs to reduce the burden of migraine in Bangladesh.

## Introduction

Migraine (without aura) was defined as recurring headache disorder that manifest in the form of attack, last 4–72 hours, unilateral, have a pulsating quality, moderate to severe in intensity, aggravated by routine physical activity and are associated with nausea or vomiting, photophobia and phonophobia [1]. It is a chronic neurovascular condition which occurs up to 15% of adult population in the Western world [2]. The burden of this disease is huge, as during the episodic attack, 90% of them experienced moderate to severe pain, 75% had some sort of disability, and 35% were confined to bed [3,4]. It is regarded as the 20^th^ leading cause of years lived with disability (YLDs) at global level and is the 9^th^ leading cause of disability in women [1]. It is one of the most common forms among the headache disorders affecting the private, social and work life of those afflicted. Repeated headache attacks and constant fear of the next, damage family life, social life and employment. It is mostly affects people of working age, but does trouble children as well. There is a dearth in population-based studies with regard to the burden of migraine in Bangladesh. One study reported that migraine is responsible for 26% of all headaches in Bangladesh [5].

Stress, smoking, pattern of sleep, weather change, missing a meal, bright light, certain food and alcohol consumption have been reported as major triggers of migraine [6-8]. Several studies demonstrated the relationship between smoking and migraine with inconsistent results [2,7-15]. However, it has not been examined if smokeless tobacco, which is highly prevalent in South Asian countries [16], is associated with migraine or not. In this region, smoked tobacco includes cigarettes, bidi (hand-rolled cigarette), hukka (water pipe for consuming smoked tobacco), while smokeless tobacco includes dried tobacco (dried tobacco leaf with betel leaf), zarda (mixture of dried tobacco leaf, spices and vegetable dyes), and gul (tobacco powder). The use of smoked tobacco in Bangladesh is much higher in males (44.7%) than females (1.5%); however, smokeless tobacco use is almost similar in both males (26.4%) and females (27.9%) [17,18].

The purpose of this study was to examine the relationship of both smoking and smokeless tobacco with migraine using a hospital-based case–control study design in Dhaka, Bangladesh.

## Methods

### Cases

Cases were 138 clinically confirmed migraine patients attended the neurology outpatient department (OPD) of Bangabandhu Sheikh Mujib Medical University (BSMMU) - a tertiary care hospital in Dhaka. Bangladesh for follow-up visits between March and September 2010. Inclusion criteria were: (1) migraine without aura diagnosed according to the criteria of the International Headache Society (IHS) [19], and (2) not suffering from known case of psychiatric illness, hypertension, hypothyroidism, or irregular menstruation. Pregnant women were not included in the study.

### Controls

Controls were 276 attendants of the cases. Two controls were matched to each case by gender and age (± 5 years). Inclusion criteria were: (1) family relative of the patient, (2) person without any history of migraine, (3) not suffering from psychiatric illness, hypertension, hypothyroidism, irregular menstruation, and (4) not pregnant.

### Data collection

Patients/attendants aged 12 years or above were included in the study. Data were collected using an interviewer-administered semi-structured questionnaire which was developed after intensive literature review and consultation with experts. The questionnaire had three parts; socio-demographic characteristics, tobacco use, and other possible risk factors (insufficient sleep, alcohol use, and mental stress). It was piloted among 15 migraine patients and 15 controls and necessary amendments were made according to the responses and suggestions of the interviewers. The respondents those who consumed any form of tobacco (smoked or smokeless) almost regularly during at least for the last six months were considered as tobacco user. The respondents were asked whether they consumed any alcoholic drink in the last two weeks. Insufficient sleep was considered as sleeping less than eight hours per day [11]. Mental stress was assessed based on the subjective judgment of the respondents. Before data collection, informed written consent was taken from the respondents. Objectives, procedure, risks, and benefits of participation in the study were included in the informed consent sheet. The participants were ensured that participation was voluntary and they were free to withdraw themselves at any time without any unwanted consequences. Moreover, confidentiality of collected data was maintained using a code for each respondent. Privacy of the participants was also maintained during data collection by interviewing into a separate room. A female health care provider was present during the interview of female respondents. The study was reviewed and approved by the Ethical Review Committee of National Institute of Preventive and Social Medicine (NIPSOM), Dhaka.

### Statistical analyses

Statistical analyses were performed with the Statistical Package for the Social Science, version 18.0 (SPSS, Chicago, IL, USA). Student's t test for continuous variables and the chi square test for categorical variables were used in the assessment of differences between the two groups when appropriate. All the statistical tests were two-tailed and *P* values <0.05 were considered as statistically significant. Exposure variables and confounders were screened for inclusion in an initial multivariable conditional logistic regression model. Candidate variables with *P* values <0.05 were included in the multivariable logistic regression model. Separate logistic regression models were built based on the type of tobacco use (nonusers, smokeless tobacco users, smokers) and frequency of tobacco use (without smokeless tobacco users).

## Results

Comparative features of cases and controls are listed in Table 1. The ages of cases and controls were identical. Cases and controls were similar with regard to education, marital status, income, and occupational status, but the former group had larger family size. More cases were smokeless tobacco users and smokers compared to controls. Among the cases, 52.8% were overall tobacco users, 24.6% were only smokers, 15.9% only smokeless tobacco users, and 12.3% used both. The respective figures among controls were 14.5%, 7.2%, 6.9%, and 0.4% (*P* <0.001 for all). Alcohol drinking, insufficient sleep and mental stress were more prevalent among cases compared to controls.

**Table 1:** Comparative features of cases and controls

	**Cases**	**Controls**	***P *value**
**Age, mean (±SD), yrs**	27.7 (8.9)	27.7 (8.9)	0.994
**Gender**			1.000
Male	46 (33.3)	92 (33.3)	
Female	92 (66.7)	184 (66.7)	
**Marital status**			0.943
Married	84 (60.9)	167 (60.5)	
Unmarried	54 (39.1)	109 (39.5)	
**Years of schooling, mean (±SD), yrs**	10.7 (4.9)	10.8 (4.7)	0.882
**Occupational status**			0.459
Unemployed	96 (69.6)	182 (65.9)	
Employed	42 (30.4)	94 (34.1)	
**Number of family member, mean (±SD)**	5.0 (1.4)	4.3 (1.2)	<0.001
**Monthly household income, mean (±SD) in BDT**	15275.3 (8382.0)	14420.2 (7066.7)	0.304
**Tobacco use**			<0.001
Non users	65 (47.1)	236 (85.5)	
Smoking only	34 (24.6)	20 (7.2)	
Smokeless tobacco only	22 (15.9)	19 (6.9)	
Both smoking and smokeless	17 (12.3)	1 (0.4)	
**Smoked tobacco users**			<0.001
Non user	87 (63.0)	255 (92.4)	
Cigarette	32 (23.2)	17 (6.2)	
Bidi	14 (10.1)	3 (1.1)	
Hukka	5 (3.6)	1 (0.4)	
Smokeless tobacco users^*^			<0.001
Non users	99 (71.7)	256 (92.8)	
Betel quid with tobacco leaf	37 (26.8)	20 (7.2)	
Zarda	35 (25.4)	16 (5.8)	
Gul	6 (4.3)	2 (0.7)	
**Number of cigarette/bidi/hukka per day**			<0.001
Non user	87 (63.0)	255 (92.4)	
1-5	8 (5.8)	5 (1.8)	
6-10	16 (11.6)	7 (2.5)	
>10	27 (19.6)	9 (3.3)	
**Alcohol use**	19 (13.8)	4 (1.4)	<0.001
**Insufficient sleep**	75 (54.3)	95 (34.4)	<0.001
**Mental stress**	93 (67.4)	89 (32.2)	<0.001

Data are mean (SD, standard deviation) or n (%).

Abbreviations: BDT, Bangladeshi Taka; 1 USD = 84 Taka.

^*^ Simultaneous consumption of >1 type of tobacco is common among smokeless tobacco users in Bangladesh.

The conditional logistic regression analysis found that migraine had higher odds of exposure to smoked tobacco use, smokeless tobacco use, and both compared to control after adjusting for confounding variables (alcohol drinking, insufficient sleep, mental stress, and number of family members); adjusted odds ratio (aOR) was 6.6 (95% confidence interval [CI] = 2.2-19.6, *P* = 0.001), 5.8 (95%CI = 1.9-17.4, *P* = 0.001), and 54.2 (95%CI = 4.3-684.4, *P* = 0.002), respectively as shown in Table 2. The aOR of cigarette/bidi/hukka smoking for different doses was 5.5 (95%CI = 1.2-24.8, *P* = 0.027) for 1–5 times per day, 6.3 (95%CI = 1.8-21.2, *P* = 0.003) for 6–10 times per day, and 6.7 (95%CI = 1.9-23.2, *P* = 0.003) for >10 times per day relative to non users (Table 3).

**Table 2:** Odds ratios for migraine by type of tobacco use

**Tobacco use**	**Crude OR (95% CI)**	***P *value**	**Adjusted OR (95% CI)**	***P *value**
Non users	1	Ref	1	Ref
Smoked tobacco	13.3 (5.5-32.4)	<0.001	6.6 (2.2-19.6)	0.001
Smokeless tobacco	8.4 (3.3-21.3)	<0.001	5.8 (1.9-17.4)	0.001
Both Smoked tobacco and Smokeless tobacco	133.9 (15.6-1146.0)	<0.001	54.2 (4.3-684.4)	0.002

Abbreviations: OR, Odds ratio, adjusted for alcohol use, insufficient sleep, mental stress and number of family member; CI, Confidence interval.

**Table 3:** Odds ratios for migraine by frequency of smoked tobacco use

**Number of cigarette/bidi/hukka per day**	**Crude OR (95% CI)**	***P *value**	**Adjusted OR (95% CI)**	***P *value**
Non user	1	Ref	1	Ref
1-5	5.5 (1.4-21.1)	0.013	5.5 (1.2-24.8)	0.027
6-10	18.0 (6.4-50.8)	<0.001	6.3 (1.8-21.2)	0.003
>10	8.1 (2.9-22.7)	<0.001	6.7 (1.9-23.2)	0.003

Abbreviations: OR, Odds ratio, adjusted for alcohol use, insufficient sleep, mental stress and number of family member; CI, Confidence interval.

## Discussion

To our knowledge, this is the first study to examine the association between smokeless tobacco use and migraine headache. We observed that smokeless tobacco users also had comparable odds of developing migraine like that of smokers within the limits inherent in case–control studies. Our findings that odds of smokers for migraine are higher compared to controls have been already reported [9,10,12-15]. Our study adds smokeless tobacco use to the list of risk factors for migraine headaches. Together, these studies provide evidence that both smokeless tobacco users and smokers are more likely to develop migraine headache compared to their counterparts.

Review of the literature showed that the associations between smoking and migraine headaches are not consistent [2,7-15]. Our finding of strong association between migraine and smoking is in agreement with some studies [2,9,10,12-15], although several studies did not find any association between them [7,8,11]. One large descriptive study also reported some sort of relationship between smoking and migraine [20]. Chen et al. [20], based on 508 migraine cases and 3192 controls, observed that there were more smokers in migraine group compared to non-migraine group. This variation between the findings could be due to the difference in study design, sample size and study population. For example, Nazari et al. study [7] conducted among women only where smoking is rarely practiced in female gender. Fernandez-de-las-penas et al.’s study [11] did not follow the IHS classification for migraine diagnosis. Takeshima et al. study [8], on the other hand, was based on secondary data which was not primarily designed to examine the association between smoking and migraine headache. Our findings showed that there were some sort of dose–response relationship between frequency of smoking and migraine. Odds ratio of migraine headache increased among smokers with the increase in frequency of smoking per day. This feature is consistent with the findings reported by Lopez-Mesonero et al. [6] and Tietjen et al. [14].

Smokeless tobacco use is widely prevalent in South Asian countries [21]. In Bangladesh, among females, 94.7% of current tobacco users used only smokeless tobacco [17]. Like other South Asian countries, the traditional values do not widely permit women to smoke cigarettes. However, there is no such taboo against using smokeless tobacco [22]. Younger people hesitate to smoke in front of their elders; they never smoke in the presence of their parents and seniors. Smokeless tobacco is an exception. Chewing betel quid (betel leaf) with sliced betel nut and dried tobacco leaf is considered as a normal social behavior. Besides, these are considered as a symbol of hospitality in the rural areas. Even the poor would feel embarrassed if these were not offered to a guest [23]. It is a social custom to serve guests by betel quid and dried tobacco product after meal in a family or social event. As smokeless tobacco contains several hazardous compounds like that of smoked tobacco, awareness against this harmful habit is must to prevent tobacco-related health conditions [24]. Campaigns against all kinds of tobacco use involving doctors, local health care workers, medicine shop keepers, barefoot doctors, and local community leaders could be more effective than other strategies. Health hazards warnings, along with nicotine and tar level, should be labeled on packets of dried tobacco leaf. Anti-tobacco campaigns by public agencies and mass media should also include smokeless tobacco in their agenda. Any efforts to reduce the use of smokeless tobacco will not only reduce migraine but also other chronic conditions [20].

Our study has several strengths which include IHS defined migraine (without aura) diagnosis, and selection of cases and controls from the same population. Although, we were aware about the issue of careful selection of controls and we have chosen controls with no past and present history of migraine attack, possibility of significant effect measures could not be eliminated. This was the major limitation of our study. Others limitations include: 1) Since our study was a prevalent case–control study, the ORs did not exactly demonstrate the relative risk of migraine incidence, but the association with those who visited the hospital a various length of period after the first pain attack. The cases with repeated pain and longer history were likely to be sampled. 2) Our study is subject to recall bias due to its study design. 3) Our findings may have limited generalizability as our study was based on a tertiary hospital. 4) Despite multivariable adjustment technique, there could be additional potential confounders which we could not measure. 5) Unavailability of dose–response data regarding smokeless tobacco was an important shortcoming in our study. 6) We couldn’t measure mental stress by using any standard scale. 7) Finally, this prevalent case–control study prevents our ability to establish causal relationships or pathways to migraine headache as we do not know whether outcome was preceded by exposure or not. Further larger scale cohort study is needed to establish the association between tobacco consumption, specially smokeless tobacco and migraine.

## Conclusions

This study indicated that both smoking and smokeless tobaccos were associated with migraine headache, although there were several limitations. As use of smokeless tobacco is widely prevalent in Bangladesh and has become a cultural norm especially in rural area, a multi-pronged approach involving community leaders, mass media, and anti-tobacco organizations is needed to formulate tobacco use cessation program along with smoked tobacco to reduce the burden of migraine in Bangladesh.

## Competing interests

The authors declare that they have no competing interests.

## Authors’ contributions

MABS contributed in the study design, data collection, and initial draft of the manuscript. MR carried out the statistical analyses and thoroughly revised the initial draft. MHOR performed study design and statistical analyses and revised the manuscript. SH was especially involved in the migraine section and study design. HK performed risk factors related to migraine section and draft of the manuscript. JS did extensive review of the study design, statistical analyses and helped to finalize the manuscript. NH carried out final revision of the manuscript. All authors read and approved the final manuscript.
